# Effectiveness of community-based interventions for prevention and control of hypertension in sub-Saharan Africa: A systematic review

**DOI:** 10.1371/journal.pgph.0003459

**Published:** 2024-07-16

**Authors:** Endalkachew Worku Mengesha, Tadesse Dagget Tesfaye, Minyahil Tadesse Boltena, Zewdie Birhanu, Morankar Sudhakar, Kalkidan Hassen, Kiya Kedir, Firaol Mesfin, Elifaged Hailemeskel, Melat Dereje, Eskedar A. Hailegebrel, Rawleigh Howe, Finina Abebe, Yordanos Tadesse, Eshetu Girma, Fisseha Wadilo, Eyasu Alem Lake, Mistire Teshome Guta, Bereket Damtew, Adisalem Debebe, Zerihun Tariku, Demuma Amdisa, Desta Hiko, Addisu Worku, Mussie G/michael, Yoseph Gebreyohannes Abraha, Sabit Ababor Ababulgu, Netsanet Fentahun

**Affiliations:** 1 College of Medicine and Health Sciences, Bahir Dar University, Bahir Dar, Ethiopia; 2 Public Health Faculty, Institute of Health, Jimma University, Jimma, Ethiopia; 3 Armauer Hansen Research Institute, Ministry of Health, Addis Ababa, Ethiopia; 4 School of Public Health, College of Health Sciences, Addis Ababa University, Addis Ababa, Ethiopia; 5 College of Health Sciences and Medicine, Wolayita Sodo University, Sodo, Ethiopia; 6 College of Medicine and Health Sciences, Dire Dawa University, Dire Dawa, Ethiopia; 7 Knowledge Translation Directorate, Ethiopian Public Health Institute, Addis Ababa, Ethiopia; 8 Ethiopian-Evidence Based Health Care and Development Centre, A JBI Centre of Excellence, Institute of Health, Jimma University, Jimma, Ethiopia; 9 Ethiopian Knowledge Translation Centre for Health, The Ethiopian Public Health Institute (EPHI), Addis Ababa, Ethiopia; 10 Ethiopian Health Education and Promotion Professionals Association, Addis Ababa, Ethiopia; 11 Federal Ministry of Health, Addis Ababa, Ethiopia; Babcock University, NIGERIA

## Abstract

Hypertension poses a significant public health challenge in sub-Saharan Africa due to various risk factors. Community-based intervention for prevention and control of hypertension is an effective strategy to minimize the negative health outcomes. However, comprehensive systematic review evidence to inform effective community-based interventions for prevention and control of hypertension in low resource settings is lacking. This study aimed to synthesize the effectiveness of community-based interventions on prevention and control of hypertension in sub-Saharan Africa. A comprehensive search for studies was carried out on PubMed, CINAHL, Web of Science Core Collection, Embase, Scopus, and Google scholar databases. The result of the review was reported according to PRISMA guidelines. Studies published in English language were included. Two independent reviewers conducted critical appraisal of included studies and extracted the data using predefined excel sheet. Experimental, quasi experimental, cohort and analytical cross-sectional studies conducted on adults who have received community-based interventions for prevention and controls of hypertension in sub-Saharan Africa were included. In this systematic review, a total of eight studies were included, comprising of two interventional studies, two quasi-experimental studies, three cohort studies, and one comparative cross-sectional study. The interventions included health education, health promotion, home-based screening and diagnosis, as well as referral and treatment of hypertensive patients. The sample sizes ranged from 236 to 13,412 in the intervention group and 346 to 6,398 in the control group. This systematic review shows the effect of community-based interventions on reduction of systolic and diastolic blood pressure. However, the existing evidence is inconsistence and not strong enough to synthesize the effect of community-based interventions for the prevention and control of hypertension in sub-Saharan Africa. Hence, further primary studies need on the effect of community-based interventions for the prevention and control of hypertension in sub-Saharan Africa.

Systematic review registration number: PROSPERO CRD42022342823.

## Introduction

Hypertension also known as the "silent killer", is a global public health concern. It is characterized by increased force exerted by circulating blood through the walls of the major blood vessels in the body [1]. Although it often has no symptoms, the consequences are devastating that can lead to heart disease, stroke, vision loss, kidney failure and other serious complications [2, 3]. The global burden of diseases report on mortality due to non-communicable diseases indicated that hypertension is the major cause of premature death. The morbidity and mortality from hypertension for the risk population group aged 30–79 years old have doubled from 1990, with 331 million women and 317 million men affected, to 2019 with 626 million women and 652 million men affected [4].

The global prevalence of hypertension has remained relatively stable over the last three decades. Women and men having similar rates of 32% and 34%, respectively, in 2019, which is comparable to 1990 levels [5]. However, the incidence of hypertension has been increasing in the past decade [6]. In low- and middle-income countries (LMICs), over an estimated 1.28 billion adults aged 30–79 years were hypertensive [7, 8].

The highest prevalence of hypertension is found in Africa, where 46% of individuals over the age of 25 are estimated to have hypertension across all World Health Organization regions [9]. Likewise, Sub-Saharan Africa (SSA), particularly in urban areas, faces significant challenges with evidence of considerable under-diagnosis, treatment, and control of hypertension [10]. The overall burden of hypertension in SSA is high and its complications are frequent. These problems are related to poor access and availability of health systems, and poor levels of awareness and control of hypertension in the region [11, 12]. While some risk factors such as age, gender, and family history cannot be modified [13, 14], hypertension also has modifiable risk factors such as physical inactivity, urbanization, low-income status, low awareness, stress, smoking, obesity, and alcohol use [15–17]. Factors that increase the risk of developing hypertension includes genetic, acquired, environmental, and societal factors [9, 18].

Although prevention and control of hypertension is vital to minimize the risk of cardiovascular and kidney diseases [14], the prevention, detection, treatment, and control of hypertension in SSA were not well-organized. Poorly designed preventive strategies at the population level, weak healthcare systems, lack of resources, and unsustainable drug therapy are some of the reasons for suboptimal standards of care. These factors have contributed to noncompliance with prescribed medications [19]. Self-monitoring of blood pressure is recommended for the management of hypertension in patients where measurement devices are affordable. However, in low- and middle-income countries many people do not seek treatment for hypertension because it is prohibitively expensive [20, 21].

Prevention and control of hypertension can be achieved through targeted and/or population-based strategies. The first step is the diagnosis of hypertension. Once hypertension is diagnosed, effective nonpharmacological and pharmacological approaches need to be implemented to lower blood pressure. Finally, treatment must be adhered to and titrated to optimize blood pressure and cardiovascular disease risk reduction. For control of hypertension, the targeted strategy involves interventions to increase awareness, treatment, and control in individuals. Community-based strategies involve interventions designed to achieve a small reduction in blood pressure in the entire population [22, 23]. Prevention includes a wide range of interventions aimed at reducing risks or threats to health. The preventive efforts should be multifaceted including the three preventive strategies. Primary prevention aims to prevent disease or injury before it ever occurs. Secondary prevention aims to reduce the impact of a disease or injury that has already occurred. Tertiary prevention aims to soften the impact of an ongoing illness or injury that has lasting effects. For example, health promotion can be applied at all three levels of disease prevention and can be of great help in maximizing the gains from preventive behaviour [24]. For example, at the primary prevention level, educating people to practice some of the preventive behaviours, such as having a balanced diet so that they can protect themselves from developing diseases in the future. At the secondary level, educating people to visit their local health canter when they experience symptoms of illness, such as fever, so they can get early treatment for their health problems. At the tertiary level, educating people to take their medication appropriately and find ways of working towards rehabilitation from significant illness or disability [25]. Screening activities also allow detection of unaware hypertensive individuals and could provide early treatment [26–28].

Although lowering blood pressure has been shown to reduce cardiovascular morbidity and mortality, the burden of hypertension remains high in sub-Saharan African countries. It is projected that the number of people affected by hypertension and its prevalence will increase in the next decade. Effective preventive strategies, such as community-based interventions, are urgently needed, especially in less developed countries, and hypertension management must be optimized [29]. While previous systematic reviews have evaluated the impact of specific interventions on hypertension in sub-Saharan Africa [30–32], there is a paucity of evidence on the effect of community-based interventions for prevention and control of hypertension. Therefore, the aim of this systematic review is to assess the effectiveness of community-based interventions in preventing and controlling hypertension in sub-Saharan Africa, which may help inform policy makers and public health practitioners in the region.

## Review question(s)

What is the effect of community-based interventions on the prevention and control of hypertension in SSA?

### Inclusion criteria

#### Participants

- All adult population, aged 18 years and above who lived in sub-Saharan Africa

### Intervention(s)

In our preliminary search, we identified key terms related to the outcome variable, community-based interventions. The interventions such as Health education or health promotion, Screening and diagnosis, and Counselling has been selected to conduct a comprehensive search in data bases and search engines.

Health education or health promotion: A mass media awareness campaign utilizing posters, billboards, mailings, and local newspaper articles. The campaign aims to promote restricted salt intake, a diet rich in fruits and vegetables, low-fat dairy products, reduced intake of saturated fat, moderate physical activity, awareness of the negative impacts of hypertension, non-drug interventions, weight management, and tobacco cessation [33].

Screening and diagnosis: Invitation of hypertensive patients for monthly monitoring of blood pressure at a health centre, with a referral system for further medical care for those with uncontrolled hypertension [34].

Counselling: advice on non-pharmacological treatment that includes weight control, reducing alcohol, dietary salt, fat and cholesterol consumption, increasing exercise, limiting smoking and stress management [34].

#### Comparator(s)

- Usual care/Standard of care

#### Outcomes

- Primary outcome: Hypertension/ high blood pressure

#### Types of studies

- Experimental, quasi-experimental, cohort and comparative cross-sectional studies

## Methods and materials

The systematic review was performed following the JBI methodology for systematic review of effectiveness studies [35]. The protocol has been registered in PROSPERO with protocol number CRD42022342823.

### Search strategy

The search strategy involved comprehensive searching on PubMed, Embase, CINAHL, Scopus, Web of Science Core Collection, and Google Scholar search engine. In addition, studies were searched in search engines, research data bases and websites including ProQuest, WHO Global Index Medicus, and Grey Net, and Google.

A three-step search strategy was performed. An initial limited search of PubMed and CINAHL were conducted to identify articles on the topic. Second, text words from the titles and abstracts of relevant articles, as well as index terms were used to develop a comprehensive search strategy.

The key terms used for the intervention were "community based intervention" OR "community based approach" OR "community based education" OR "community based promotion" OR "community based screening" OR "population based approach" OR "population based intervention" OR "community based diagnosis" OR "population based screening" OR "population based diagnosis" OR "population based education" OR "Population-Based promotion" OR "Population-Based counselling" OR "community based counselling" OR "population based training" OR "community based training". In addition, the key terms used for the outcome include "Hypertension" OR "blood pressure” OR "arterial pressure" OR "raised blood pressure" (**S1 Text**). The search strategy includes all the identified keywords and index terms adapted for each database. Lastly, the reference list of all included studies was screened for additional studies. Only English language studies were included in the review.

### Study selection

All identified citations were uploaded into EndNote Version X8 and duplicates were removed.

**Fig 1:**
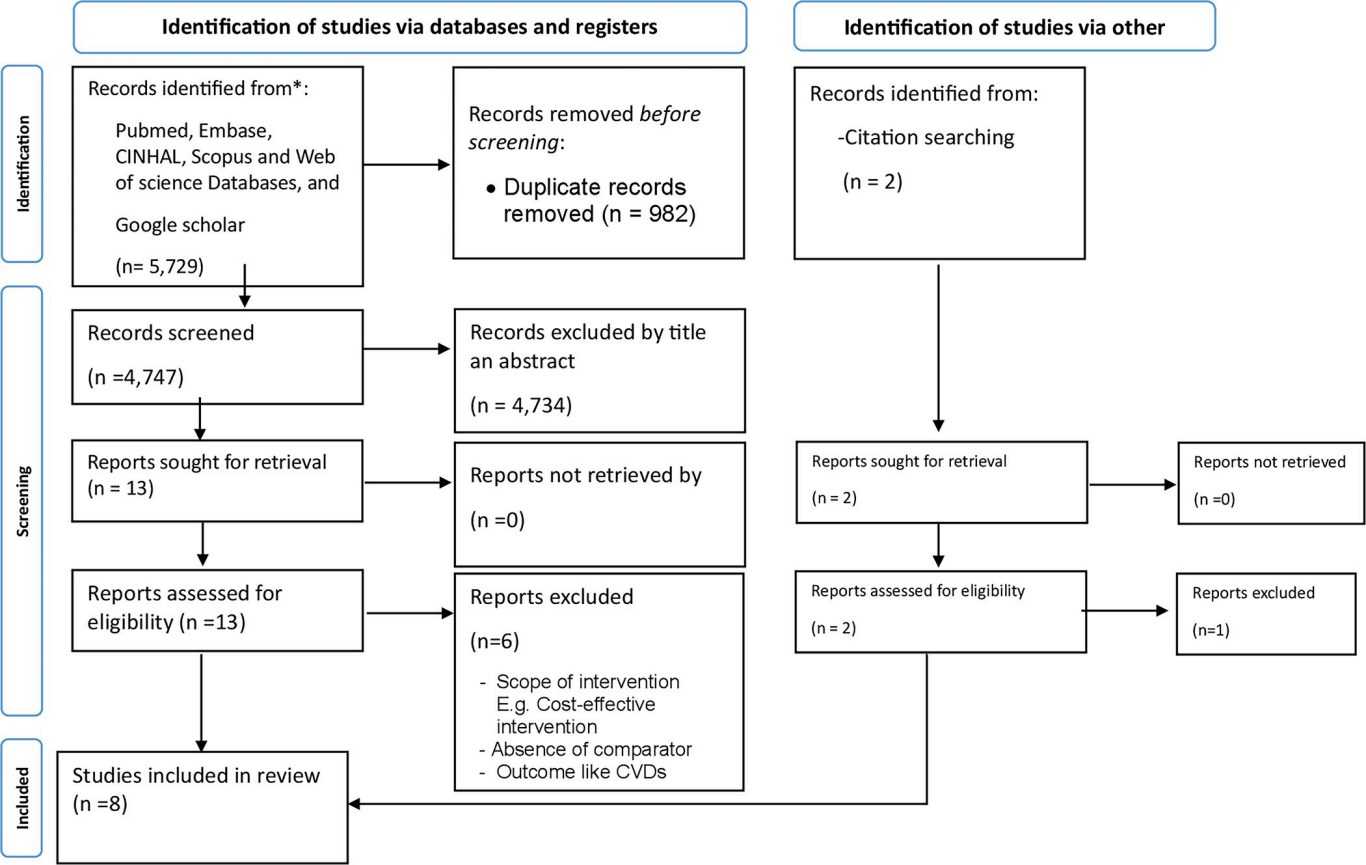
The PRISMA flow diagram [36].

Two independent reviewers (EWM & TDT) screened the titles and abstracts, and the full text of selected citations was assessed in detail against the inclusion criteria by the same reviewers. Any disagreements that arose between the reviewers at each stage of the selection process were resolved through discussion, or in consultation with an additional reviewer (NF). The complete results of the search and study inclusion process are reported in the final systematic review and presented in a Preferred Reporting Items for Systematic Reviews and Meta-analyses (PRISMA) flow diagram [36]. **(Fig 1)**.

### Assessment of methodological quality

The methodological quality of eligible studies was assessed by two independent reviewers (EWM & TDT) using the JBI standardized critical appraisal instruments for randomized control trials, quasi-experimental, cohort, and analytical cross-sectional studies [35, 36]. The results of the critical appraisal were reported in both narrative and tabular form. All studies that met the inclusion criteria, regardless of their methodological quality, underwent data extraction and synthesis.

### Data extraction

Data from the included studies in the review were independently extracted by two reviewers (EWM & TDT) using an Excel sheet adapted from the standard JBI tools [35]. The data extraction tool included information about year of publication, country, context (community-based or home-based settings), participants’ characteristics such as sample size, age, sex, type of intervention received for prevention and control of hypertension, and outcomes measured.

### Data synthesis

Descriptive data synthesis was conducted since included studies presented their findings narratively, making it implausible to estimate the pooled effect size. Moreover, the studies used different narrations of the duration, intensity, and approach of the intervention.

### Deviation from protocol

The deviation occurred due to unforeseen circumstance and has been thoroughly documented below to ensure transparency and maintain the integrity of the research process. According to our initial protocol plan, our intention was to exclusively review randomized controlled trials (RCTs) as part of our study. However, during the course of our comprehensive search, we encountered a significant scarcity of relevant studies (unforeseen circumstance) that met the criteria of RCTs. As a result, we made the decision to deviate from our protocol and include other study designs in our analysis.

The primary reason for this deviation was our belief that including studies of different designs would still provide valuable information that could contribute to our research objectives. While RCTs are considered the gold standard in clinical research due to their rigorous methodology and ability to establish causal relationships, other study designs such as observational studies can offer insights into real-world scenarios, patient experiences, and contextual factors that RCTs may not capture.

By broadening the scope of our study to encompass various study designs, we aimed to gather a more comprehensive understanding of the topic at hand. Although this decision deviated from our original protocol, it was made with the intention of maximizing the available evidence and ensuring that our analysis would be as informative and useful as possible.

Despite the inclusion of non-RCT studies, it is important to acknowledge the inherent limitations associated with these study designs, such as potential biases and confounding factors. However, by transparently reporting our departure from the initial protocol and incorporating a diverse range of evidence, we aimed to mitigate these limitations and provide a balanced assessment of the available research.

In summary, due to the limited number of RCTs identified during our search, we deviated from our original protocol and included other study designs in our review. This decision was driven by the recognition that these additional studies could still provide valuable information, albeit with certain limitations. By expanding the scope of our analysis, we aimed to enhance the comprehensiveness and applicability of our findings, ultimately contributing to a more nuanced understanding of the subject matter.

## Results

### Study selection and characteristics

The initial search yielded a total of 5,731 records (as shown in Fig 1 above). After removing duplicates and further screening by title and abstract, a total of 14 eligible studies were identified. Among these, only eight articles reach for critical appraisal and included in this systematic review. Two of them were community-based intervention studies [37, 38], two were quasi-experimental studies [33, 39], three were cohort studies [40–42], and one was comparative cross- sectional study [34].

Most of the included studies involved women in rural settings [33]. Two studies included participants with low socio-economic status such as high level of poverty, unemployment and residence in slum areas [38, 39].

Intervention modalities included health education, health promotion, home- based screening and diagnosis, referral and treatment of individuals with hypertension. The sample sizes for the intervention arm ranged from 236 to 13,412 individuals, while for the control arm it ranged from 346 to 6,398 individuals.

In one of the studies, women aged 24 years or older were found to be at a higher risk for hypertension [33]. However, in another study, women had better linkage to the care pathway [41] (**Table 1**).

**Table 1:** Study characteristics of included studies in the review.

Author, publication year	Country	Study design	Sample size	Intervention modality
Rossouw J. E., et al.,1993	South Africa	Quasi-experimental	13,412(I) 6,398(C)	Health education and promotion
Flor et al., 2020	South Africa	Quasi- experimental design		Screening and diagnosis
Pastakia et al., 2013	Kenya	Community based interventional study	236 (I) 346 (C)	Home based screening and Community based screening
Van de Vijver, et al., 2016	Kenya	Prospective intervention study	1,531 (I) 1,233 (C)	Awareness campaigns, household visits for screening, and referral and treatment of people with hypertension
Siedner et al., 2018	South Africa	A population-based cohort study	346 (I) 856 (C)	Home based screening
Kotwani et al., 2014	Uganda	prospective cohort study		Hypertension linkage to care nested within a multi-disease community health campaign
Steyn K et al., 1993	South Africa	Comparative cross-sectional design	1,077 (I) 543 (C)	Health promotion
Nikkil Sudharsanan et al., 2020	South Africa	longitudinal study (follow-up)		Home-based screening & diagnosis

### Main findings

Health education, health promotion, community-based screening, home- based screening and diagnosis were the identified intervention modalities for the prevention and control of hypertension in Sub-Sharan Africa.

### Effect of health education and health promotion for the prevention and control of hypertension

Community-based interventions such as health education program significantly reduced blood pressure of people with elevated pressure because of mainstream media campaign, billboards, mailings, posters and items in local newspapers. These interventions created awareness in the community and they were also cost effective when compared to interpersonal interventions [33]. (**S1 Table**).

### Effect of home-based, community-based screening and diagnosis for the prevention and control of hypertension

The community-based screening strategy had the potential to reach a high-risk population. Additionally, home-based screening and diagnosis has the potential for a greater linkage to the healthcare system [37] and were more likely benefit women and elders who spent more time at home [40]. Despite high rates of lost follow-up recorded in a study conducted in South Africa, the home-based screening intervention showed important reductions in systolic blood pressure for women and younger men [42].

Overall, health education, screening, and referral appointments to a health facility increased successful linkage and early visit of a health facility for hypertension management. However, perceived feeling of being well, transportation cost and inconvenience fear of being reprimanded by the clinic staff for missing a scheduled appointment, family obligations and responsibilities were the most common barriers for not linking to care [39].

A study conducted in a slum area of Kenya reported that community-based interventions such as awareness campaigns, screening, referral, and treatment of people with hypertension had no effect on the reduction of hypertension [38] **(S1 Table).**

## Discussion

The aim of this systematic review was to evaluate the effectiveness of community-based interventions for the prevention and control of hypertension in SSA. Based on our review, community-based interventions including health education intervention that used mass media campaigns, such as posters, billboards, mailings, and items in local newspapers, were found to significantly reduce blood pressure [33, 37]. This is consistent with a previous systematic review that reported positive effects of health promotion interventions on the reduction of blood pressure in India and Indonesia [43]. Other studies conducted in developing countries indicated that the implementation of community-based programs has great potential to control hypertension. Particularly, active screening, and diagnosis in Cuban system could serve as an example for other low- and middle-income countries [44, 45].

The community-based screening of hypertension has been found to improve access to life-saving care for high-risk populations [46]. Implementation of home-based screening and diagnosis of hypertension is key for linkage of the care pathway of hypertensive older population including women to the healthcare system [37, 40]. These findings are consistent with the WHO’s recommendations on the response to non-communicable diseases [47].

Social mobilization and women empowerment as change agents in families and communities through engaging human rights groups, organizations (like faith-based, labour, children focused, intergovernmental and nongovernmental organizations), adolescents, youth, adults, elderly, women, patients and people with disabilities, indigenous peoples, civil society, academia, media and the private sector had a significant contribution for prevention and control of hypertension [47].

Screening and referral appointment to a health facility increased successful linkage and early visit of a health facility for hypertension management. Perceived feeling of being well, transportation cost and difficulty/inconvenience, fear of being reprimanded by the clinic staff for missing a scheduled appointment, family obligations and responsibilities at work impeding clinic visit were the most barriers for poor linkage of hypertension cases standard of care [41]. Non-pharmacological interventions such as community-based health education and task-shifting strategies for home visits were effective strategies to improve blood pressure control in SSA [48].

Despite the aforementioned findings favouring blood pressure reduction, a study conducted in a slum area of Kenya reported that community-based interventions, such as awareness campaigns, screening, referral, and treatment of people with hypertension had no effect on reduction of hypertension [38]. This might be due to low levels of health education; poverty and limited access to affordable and nutritious food in slum areas [49].

### Strength and limitation of the study

The strength of this systematic review was the use of five data bases and one search engine for searching primary studies, which made our search strategy comprehensive. In addition, this review highlighted the potential benefit of community-based interventions on the prevention and control of hypertension. A research question has also a government concern that was co-designed by the non-communicable diseases’ directorate at the Ethiopian Ministry of Health. In spite of the above strengths, drawing of conclusions about effectiveness of community-based interventions were challenging due to heterogeneity in intervention modality, study design, intensity and duration of interventions in the included studies, which limited us from performing a meta-analysis. And also, deviation from protocol (described in method section), restrictions of the literature published in English language were the limitations of this study.

### Implications for practice

Community-based interventions such as; health education, health promotion, community-based screening, home- based screening and diagnosis had positive effect on reduction of systolic and diastolic blood pressure. Hence, program implementers may consider these interventions for prevention and control of hypertension. However, the evidence was not strong enough for designing a program based on these interventions alone.

### Implications for research

This review found limited records with varying intervention modalities, and most of the included studies focused on women in rural settings. The existing evidences are inconsistent and not strong enough to synthesize the effect of community-based interventions for the prevention and control of hypertension in sub-Saharan Africa. Therefore, further primary studies are needed to investigate the effect of community-based interventions for the prevention and control of hypertension in sub-Saharan Africa.

## Conclusion

Most of the studies reviewed suggest that community-based interventions could have potential benefits for the prevention and control of hypertension in sub-Saharan Africa. Health promotion, community- and home- based screening and diagnosis were mentioned as cost-effective intervention modalities. Stakeholders involved in non-communicable disease control shall seek for up-to-date evidence from primary studies.

## Supporting information

S1 Checklist Critical appraisal for various studies. (DOCX)

S2 Checklist PRISMA checklist. (PDF)

S1 Text This is the S1 search strategy on various databases and search engine. (DOCX)

S1 Table Table depicting key findings on the effect of community-based interventions on prevention and control of hypertension. (DOCX)

S1 Data (XLSX)
