# Supplementary material for: Effectiveness of community-based interventions for prevention and control of hypertension in sub-Saharan Africa: A systematic review
Source: PLOS Glob Public Health. 2024 Jul 16;4(7):e0003459. doi: 10.1371/journal.pgph.0003459 (PMC11251591; doi:10.1371/journal.pgph.0003459)
Supplement: S1 Checklist — (DOCX) [file pgph.0003459.s001.docx]

## S2 Critical appraisals Checklists

# Table 1: Critical appraisal for included quasi-experimental studies

| **First author, year of publication** | **Q1** | **Q2** | **Q3** | **Q4** | **Q5** | **Q6** | **Q7** | **Q8** | **Q9** |
| --- | --- | --- | --- | --- | --- | --- | --- | --- | --- |
| Rossouw et al.,1993 | Y | Y | Y | Y | Y | N | Y | Y | Y |
| Flor et al., 2020 | Y | Y | Y | N | Y | U | Y | Y | Y |
| **Number of studies that achieved compliance** | 2 | 2 | 2 | 1 | 2 | 0 | 2 | 2 | 2 |

The following criteria were from the JBI Critical Appraisal Checklist for quasi-experimental studies. (1) Is it clear in the study what the ‘cause’ is and what is the ‘effect’ (i.e. there is no confusion about which variable comes first)? (2) Were the participants included in any comparisons similar? (3) Were the participants included in any comparisons receiving similar treatment/care, other than the exposure or intervention of interest? (4) Was there a control group? (5) Were there multiple measurements of the outcome both pre and post the intervention/ exposure? (6) Was follow up complete and if not, were differences between groups in terms of their follow up adequately described and analyzed? (7) Were the outcomes of participants included in any comparisons measured in the same way? (8) Were outcomes measured in a reliable way? (9) Was appropriate statistical analysis used? Each item was rated Y = Yes, N = No or U = Unclear. Unclear was awarded where not enough information was provided.

# Table 2: Critical appraisal for included randomized controlled trials

| **First author, year of publication** | **Q1** | **Q2** | **Q3** | **Q4** | **Q5** | **Q6** | **Q7** | **Q8** | **Q9** | **Q10** | **Q11** | **Q12** | **Q13** |
| --- | --- | --- | --- | --- | --- | --- | --- | --- | --- | --- | --- | --- | --- |
| Pastakia et al., 2013 | N | Y | Y | Y | Y | N | Y | Y | Y | Y | Y | Y | Y |
| van de Vijver, et al., 2016 | Y | Y | Y | U | Y | U | Y | Y | Y | Y | Y | Y | Y |
| **Number of studies that achieved compliance** | 1 | 2 | 2 | 1 | 2 | 0 | 2 | 2 | 2 | 2 | 2 | 2 | 2 |

The following criteria were from the JBI Critical Appraisal Checklist for randomized controlled trials. (1) Was true randomization used for assignment of participants to treatment groups? (2) Was allocation to treatment groups concealed? (3) Were treatment groups similar at the baseline? (4) Were participants blind to treatment assignment? (5) Were those delivering treatment blind to treatment assignment? (6) Were outcomes assessors blind to treatment assignment? (7) Were treatment groups treated identically other than the intervention of interest? (8) Was follow up complete and if not, were differences between groups in terms of their follow up adequately described and analyzed? (9) Were participants analyzed in the groups to which they were randomized? (10) Were outcomes measured in the same way for treatment groups? (11) Were outcomes measured in a reliable way? (12) Was appropriate statistical analysis used? (13) Was the trial design appropriate and any deviations from the standard RCT design (individual randomization, parallel groups) accounted for in the conduct and analysis of the trial? Each item was rated Y = Yes, N = No or U = Unclear. Unclear was awarded where not enough information was provided.

# Table 3: Critical appraisal for included cohort studies

| **First author, year of publication** | **Q1** | **Q2** | **Q3** | **Q4** | **Q5** | **Q6** | **Q7** | **Q8** | **Q9** | **Q10** | **Q11** |
| --- | --- | --- | --- | --- | --- | --- | --- | --- | --- | --- | --- |
| Siedner et al., 2018 | N | Y | Y | Y | Y | N | Y | Y | Y | Y | Y |
| Kotwani et al., 2014 | Y | Y | Y | N | Y | U | Y | Y | Y | Y | Y |
| Nikkil Sudharsanan et al., 2020 | Y | Y | Y | N | Y | U | Y | Y | Y | Y | Y |
| **Number of studies that achieved compliance** | 2 | 3 | 3 | 1 | 3 | 0 | 3 | 3 | 3 | 3 | 3 |

The following criteria were from the JBI Critical Appraisal Checklist for cohort studies (1) Were the two groups similar and recruited from the same population? (2) Were the exposures measured similarly to assign people to both exposed and unexposed groups? (3) Was the exposure measured in a valid and reliable way? (4) Were confounding factors identified? (5) Were strategies to deal with confounding factors stated? (6) Were the groups/participants free of the outcome at the start of the study (or at the moment of exposure)? (7) Were the outcomes measured in a valid and reliable way? (8) Was the follow up time reported and sufficient to be long enough for outcomes to occur? (9) Was follow up complete, and if not, were the reasons to loss to follow up described and explored? (10) Were strategies to address incomplete follow up utilized? (11) Was appropriate statistical analysis used? Each item was rated Y = Yes, N = No or U = Unclear. Unclear was awarded where not enough information was provided.

# Table 4: Critical appraisal for included analytical cross sectional studies

| **First author and year of publication** | **Q1** | **Q2** | **Q3** | **Q4** | **Q5** | **Q6** | **Q7** | **Q8** |
| --- | --- | --- | --- | --- | --- | --- | --- | --- |
| Steyn K et al., 1993 | N | Y | Y | Y | U | N | Y | Y |

The following criteria were from the JBI Critical Appraisal Checklist for analytical cross sectional studies. (1) Were the criteria for inclusion in the sample clearly defined? (2) Were the study subjects and the setting described in detail? (3) Was the exposure measured in a valid and reliable way? (4) Were objective, standard criteria used for measurement of the condition? (5) Were confounding factors identified? (6) Were strategies to deal with confounding factors stated? (7) Were the outcomes measured in a valid and reliable way? (8) Was appropriate statistical analysis used? Each item was rated Y = Yes, N = No or U = Unclear. Unclear was awarded where not enough information was provided.
