# Supplementary material for: Effectiveness of community-based interventions for prevention and control of hypertension in sub-Saharan Africa: A systematic review
Source: PLOS Glob Public Health. 2024 Jul 16;4(7):e0003459. doi: 10.1371/journal.pgph.0003459 (PMC11251591; doi:10.1371/journal.pgph.0003459)
Supplement: S1 Text — (DOCX) [file pgph.0003459.s003.docx]

# S1 Appendices

## Appendix I: Search strategy on Pubmed database

### Search conducted on October 05, 2022.

| **Search** | **Query** | **Records retrieved** |
| --- | --- | --- |
| #1 | "community based intervention*" OR "community based approach*" OR "community based education*" OR "community based promotion*" OR "community based screening" OR "population based approach*" OR "population based intervention*" OR "community based diagnosis*" OR "population based screening" OR "population based diagnosis*" OR "population based education*" OR ("Population-Based" AND "promotion*") OR ("Population-Based" AND "counseling") OR "community based counseling" OR "population based training*" OR "community based training*" | 13,318 |
| #2 | "Hypertension"[MeSH Terms] OR "Hypertension" OR "blood pressure"[MeSH Terms] OR "blood pressure determination"[MeSH Terms] OR "arterial pressure"[MeSH Terms] OR "blood pressure" OR "raised blood pressure" | 894,579 |
| #3 | "Sub-Saharan Africa" OR "Africa, Sub-Saharan" OR "Subsaharan Africa" OR "Africa South of the Sahara"[MeSH Terms] OR Angola OR "Cape Verde" OR "Cabo Verde" OR Cameroon OR Comoros OR Congo OR "Côte d'Ivoire" OR Djibouti OR Egypt OR Eswatini OR Ghana OR Kenya OR Lesotho OR Mauritania OR Nigeria OR "São Tomé and Principe" OR Senegal OR Sudan OR Zambia OR Benin OR "Burkina Faso" OR Burundi OR "Central African Republic" OR Chad OR Eritrea OR Ethiopia OR Gambia OR Guinea OR "Guinea-Bissau" OR Liberia OR Madagascar OR Malawi OR Mali OR Mozambique OR Niger OR Rwanda OR "Sierra Leone" OR Somalia OR "South Sudan" OR "South Africa" OR Tanzania OR Togo OR Uganda | 763,149 |
| #4(#1 AND #2 AND #3) | (("community based intervention*" OR "community based approach*" OR "community based education*" OR "community based promotion*" OR "community based screening" OR "population based approach*" OR "population based intervention*" OR "community based diagnosis*" OR "population based screening" OR "population based diagnosis*" OR "population based education*" OR ("Population-Based" AND "promotion*") OR ("Population-Based" AND "counseling") OR "community based counseling" OR "population based training*" OR "community based training*") AND ("Hypertension"[MeSH Terms] OR "Hypertension" OR "blood pressure"[MeSH Terms] OR "blood pressure determination"[MeSH Terms] OR "arterial pressure"[MeSH Terms] OR "blood pressure" OR "raised blood pressure")) AND ("Sub-Saharan Africa" OR "Africa, Sub-Saharan" OR "Subsaharan Africa" OR "Africa South of the Sahara"[MeSH Terms] OR Angola OR "Cape Verde" OR "Cabo Verde" OR Cameroon OR Comoros OR Congo OR "Côte d'Ivoire" OR Djibouti OR Egypt OR Eswatini OR Ghana OR Kenya OR Lesotho OR Mauritania OR Nigeria OR "São Tomé and Principe" OR Senegal OR Sudan OR Zambia OR Benin OR "Burkina Faso" OR Burundi OR "Central African Republic" OR Chad OR Eritrea OR Ethiopia OR Gambia OR Guinea OR "Guinea-Bissau" OR Liberia OR Madagascar OR Malawi OR Mali OR Mozambique OR Niger OR Rwanda OR "Sierra Leone" OR Somalia OR "South Sudan" OR "South Africa" OR Tanzania OR Togo OR Uganda) | 81 |

## Appendix II: Search strategy on Embase (Elsevier) database

### Search date: October 05, 2022

| **Search #** | **Query** | **Records Retrieved** |
| --- | --- | --- |
| 1 | 'community based intervention'/exp OR 'community based intervention' OR 'community based approach' OR (('community'/exp OR community) AND based AND approach) OR 'community based education' OR (('community'/exp OR community) AND based AND ('education'/exp OR education)) OR 'community based diagnosis' OR (('community'/exp OR community) AND based AND ('diagnosis'/exp OR diagnosis)) OR 'community based screening' OR (('community'/exp OR community) AND based AND ('screening'/exp OR screening)) OR 'population based approach' OR (('population'/exp OR population) AND based AND approach) OR 'population based intervention' OR (('population'/exp OR population) AND based AND ('intervention'/exp OR intervention)) OR 'population based education' OR (('population'/exp OR population) AND based AND ('education'/exp OR education)) OR 'population based screening' OR (('population'/exp OR population) AND based AND ('screening'/exp OR screening)) | 401,314 |
| 2 | 'hypertension'/exp OR hypertension | 1,232,083 |
| 3 | 'africa south of the sahara'/exp OR 'sub-saharan africa'/exp OR 'sub-saharan africa' OR ('sub saharan' AND ('africa'/exp OR africa)) | 319,138 |
| 4 | 1 AND 2 AND 3 | 833 |

## Appendix III: Search strategy on CINAHL database

### Search conducted on October 06, 2022.

| **Search** | **Query** | **Records retrieved** |
| --- | --- | --- |
| S1 | TI "community based intervention*" OR "community based approach*" OR "community based education*" OR "community based promotion*" OR "community based screening" OR "population based approach*" OR "population based intervention*" OR "community based diagnosis*" OR "population based screening" OR "population based diagnosis*" OR "population based education*" OR "Population-Based promotion*" OR "Population-Based counseling*" OR "community based counseling" OR "population based training*" OR "community based training*" | 1,086 |
| S2 | TI "Hypertension*" OR "blood pressure*" OR "blood pressure determination*" OR "arterial pressure*" OR "blood pressure*" OR "raised blood pressure*" | 58,226 |
| S3 | TI "Sub-Saharan Africa" OR "Africa, Sub-Saharan" OR "Subsaharan Africa" OR "Africa South of the Sahara" OR Angola OR "Cape Verde" OR "Cabo Verde" OR Cameroon OR Comoros OR Congo OR "Côte d'Ivoire" OR Djibouti OR Egypt OR Eswatini OR Ghana OR Kenya OR Lesotho OR Mauritania OR Nigeria OR "São Tomé and Principe" OR Senegal OR Sudan OR Zambia OR Benin OR "Burkina Faso" OR Burundi OR "Central African Republic" OR Chad OR Eritrea OR Ethiopia OR Gambia OR Guinea OR "Guinea-Bissau" OR Liberia OR Madagascar OR Malawi OR Mali OR Mozambique OR Niger OR Rwanda OR "Sierra Leone" OR Somalia OR "South Sudan" OR "South Africa" OR Tanzania OR Togo OR Uganda | 52,425 |
| S4 | S1 AND S2 AND S3 | 3 |

## Appendix IV: Search strategy on Scopus database

### Search conducted on October 06, 2022.

| **Search** | **Query** | **Records retrieved** |
| --- | --- | --- |
| S1 | "community based intervention" OR "community based approach" OR "community based education" OR "community based promotion" OR "community based screening" OR "population based approach" OR "population based intervention" OR "community based diagnosis" OR "population based screening" OR "population based diagnosis" OR "population based education" OR "population-based promotion" OR "population-based counseling" OR "community based counseling" OR "population based training" OR "community based training" | 59,431 |
| S2 | "hypertension" OR "blood pressure" OR "blood pressure determination" OR "arterial pressure" OR "blood pressure" OR "raised blood pressure" | 2,412,250 |
| S3 | "sub-saharan africa" OR "africa, sub-saharan" OR "subsaharan africa" OR "africa south of the sahara" OR angola OR "cape verde" OR "cabo verde" OR cameroon OR comoros OR congo OR "côte d'ivoire" OR djibouti OR egypt OR eswatini OR ghana OR kenya OR lesotho OR mauritania OR nigeria OR "são tomé and principe" OR senegal OR sudan OR zambia OR benin OR "burkina faso" OR burundi OR "central african republic" OR chad OR eritrea OR ethiopia OR gambia OR guinea OR "guinea-bissau" OR liberia OR madagascar OR malawi OR mali OR mozambique OR niger OR rwanda OR "sierra leone" OR somalia OR "south sudan" OR "south africa" OR tanzania OR togo OR uganda | 4,510,898 |
| S4 | S1 AND S2 AND S3  ( "community based intervention" OR "community based approach" OR "community based education" OR "community based promotion" OR "community based screening" OR "population based approach" OR "population based intervention" OR "community based diagnosis" OR "population based screening" OR "population based diagnosis" OR "population based education" OR "population-based promotion" OR "population-based counselling" OR "community based counselling" OR "population based training" OR "community based training" ) AND ( "hypertension" OR "blood pressure" OR "blood pressure determination" OR "arterial pressure" OR "blood pressure" OR "raised blood pressure" ) AND ( "sub-saharan africa" OR "africa, sub-saharan" OR "subsaharan africa" OR "africa south of the sahara" OR angola OR "cape verde" OR "cabo verde" OR cameroon OR comoros OR congo OR "côte d'ivoire" OR djibouti OR egypt OR eswatini OR ghana OR kenya OR lesotho OR mauritania OR nigeria OR "são tomé and principe" OR senegal OR sudan OR zambia OR benin OR "burkina faso" OR burundi OR "central african republic" OR chad OR eritrea OR ethiopia OR gambia OR guinea OR "guinea-bissau" OR liberia OR madagascar OR malawi OR mali OR mozambique OR niger OR rwanda OR "sierra leone" OR somalia OR "south sudan" OR "south africa" OR tanzania OR togo OR uganda ) | 1,713 |

## Appendix V: Search strategy on Web of Sciences database

### Search conducted on October 06, 2022.

| **Search** | **Query** | **Records retrieved** |
| --- | --- | --- |
| S1 | TS=(community based approach* OR community based intervention* OR education* OR training* OR promotion* OR screening* OR diagnosis* OR counseling* OR population based intervention*) | 5,036,844 |
| S2 | TS=(Hypertension* OR blood pressure* OR blood pressure determination* OR arterial pressure* OR blood pressure* OR raised blood pressure*) | 830,142 |
| S3 | TS=(Sub-Saharan Africa OR Africa, Sub-Saharan OR Subsaharan Africa OR Africa South of the Sahara OR Angola OR Cape Verde OR Cabo Verde OR Cameroon OR Comoros OR Congo OR Côte d'Ivoire OR Djibouti OR Egypt OR Eswatini OR Ghana OR Kenya OR Lesotho OR Mauritania OR Nigeria OR São Tomé and Principe OR Senegal OR Sudan OR Zambia OR Benin OR Burkina Faso OR Burundi OR Central African Republic OR Chad OR Eritrea OR Ethiopia OR Gambia OR Guinea OR Guinea-Bissau OR Liberia OR Madagascar OR Malawi OR Mali OR Mozambique OR Niger OR Rwanda OR Sierra Leone OR Somalia OR South Sudan OR South Africa OR Tanzania OR Togo OR Uganda) | 802,808 |
| S4 | S1 AND S2 AND S3 | 2,645 |

## Appendix VI: Search strategy on Google Scholar

### Search conducted on October 06, 2022.

| **Search** | **Query** | **Records retrieved** |
| --- | --- | --- |
| S1 | allintitle: "community based" AND "hypertension" | 454 |

## Annex VII: Critical appraisals

# Table 1: Critical appraisal for included quasi-experimental studies

| **First author, year of publication** | **Q1** | **Q2** | **Q3** | **Q4** | **Q5** | **Q6** | **Q7** | **Q8** | **Q9** |
| --- | --- | --- | --- | --- | --- | --- | --- | --- | --- |
| Rossouw et al.,1993 | Y | Y | Y | Y | Y | N | Y | Y | Y |
| Flor et al., 2020 | Y | Y | Y | N | Y | U | Y | Y | Y |
| **Number of studies that achieved compliance** | 2 | 2 | 2 | 1 | 2 | 0 | 2 | 2 | 2 |

The following criteria were from the JBI Critical Appraisal Checklist for quasi-experimental studies. (1) Is it clear in the study what the ‘cause’ is and what is the ‘effect’ (i.e. there is no confusion about which variable comes first)? (2) Were the participants included in any comparisons similar? (3) Were the participants included in any comparisons receiving similar treatment/care, other than the exposure or intervention of interest? (4) Was there a control group? (5) Were there multiple measurements of the outcome both pre and post the intervention/ exposure? (6) Was follow up complete and if not, were differences between groups in terms of their follow up adequately described and analyzed? (7) Were the outcomes of participants included in any comparisons measured in the same way? (8) Were outcomes measured in a reliable way? (9) Was appropriate statistical analysis used? Each item was rated Y = Yes, N = No or U = Unclear. Unclear was awarded where not enough information was provided.

# Table 2: Critical appraisal for included randomized controlled trials

| **First author, year of publication** | **Q1** | **Q2** | **Q3** | **Q4** | **Q5** | **Q6** | **Q7** | **Q8** | **Q9** | **Q10** | **Q11** | **Q12** | **Q13** |
| --- | --- | --- | --- | --- | --- | --- | --- | --- | --- | --- | --- | --- | --- |
| Pastakia et al., 2013 | N | Y | Y | Y | Y | N | Y | Y | Y | Y | Y | Y | Y |
| van de Vijver, et al., 2016 | Y | Y | Y | U | Y | U | Y | Y | Y | Y | Y | Y | Y |
| **Number of studies that achieved compliance** | 1 | 2 | 2 | 1 | 2 | 0 | 2 | 2 | 2 | 2 | 2 | 2 | 2 |

The following criteria were from the JBI Critical Appraisal Checklist for randomized controlled trials. (1) Was true randomization used for assignment of participants to treatment groups? (2) Was allocation to treatment groups concealed? (3) Were treatment groups similar at the baseline? (4) Were participants blind to treatment assignment? (5) Were those delivering treatment blind to treatment assignment? (6) Were outcomes assessors blind to treatment assignment? (7) Were treatment groups treated identically other than the intervention of interest? (8) Was follow up complete and if not, were differences between groups in terms of their follow up adequately described and analyzed? (9) Were participants analyzed in the groups to which they were randomized? (10) Were outcomes measured in the same way for treatment groups? (11) Were outcomes measured in a reliable way? (12) Was appropriate statistical analysis used? (13) Was the trial design appropriate and any deviations from the standard RCT design (individual randomization, parallel groups) accounted for in the conduct and analysis of the trial? Each item was rated Y = Yes, N = No or U = Unclear. Unclear was awarded where not enough information was provided.

# Table 3: Critical appraisal for included cohort studies

| **First author, year of publication** | **Q1** | **Q2** | **Q3** | **Q4** | **Q5** | **Q6** | **Q7** | **Q8** | **Q9** | **Q10** | **Q11** |
| --- | --- | --- | --- | --- | --- | --- | --- | --- | --- | --- | --- |
| Siedner et al., 2018 | N | Y | Y | Y | Y | N | Y | Y | Y | Y | Y |
| Kotwani et al., 2014 | Y | Y | Y | N | Y | U | Y | Y | Y | Y | Y |
| Nikkil Sudharsanan et al., 2020 | Y | Y | Y | N | Y | U | Y | Y | Y | Y | Y |
| **Number of studies that achieved compliance** | 2 | 3 | 3 | 1 | 3 | 0 | 3 | 3 | 3 | 3 | 3 |

The following criteria were from the JBI Critical Appraisal Checklist for cohort studies (1) Were the two groups similar and recruited from the same population? (2) Were the exposures measured similarly to assign people to both exposed and unexposed groups? (3) Was the exposure measured in a valid and reliable way? (4) Were confounding factors identified? (5) Were strategies to deal with confounding factors stated? (6) Were the groups/participants free of the outcome at the start of the study (or at the moment of exposure)? (7) Were the outcomes measured in a valid and reliable way? (8) Was the follow up time reported and sufficient to be long enough for outcomes to occur? (9) Was follow up complete, and if not, were the reasons to loss to follow up described and explored? (10) Were strategies to address incomplete follow up utilized? (11) Was appropriate statistical analysis used? Each item was rated Y = Yes, N = No or U = Unclear. Unclear was awarded where not enough information was provided.

# Table 4: Critical appraisal for included analytical cross sectional studies

| **First author and year of publication** | **Q1** | **Q2** | **Q3** | **Q4** | **Q5** | **Q6** | **Q7** | **Q8** |
| --- | --- | --- | --- | --- | --- | --- | --- | --- |
| Steyn K et al., 1993 | N | Y | Y | Y | U | N | Y | Y |

The following criteria were from the JBI Critical Appraisal Checklist for analytical cross sectional studies. (1) Were the criteria for inclusion in the sample clearly defined? (2) Were the study subjects and the setting described in detail? (3) Was the exposure measured in a valid and reliable way? (4) Were objective, standard criteria used for measurement of the condition? (5) Were confounding factors identified? (6) Were strategies to deal with confounding factors stated? (7) Were the outcomes measured in a valid and reliable way? (8) Was appropriate statistical analysis used? Each item was rated Y = Yes, N = No or U = Unclear. Unclear was awarded where not enough information was provided.
