# Supplementary material for: Effectiveness of community-based interventions for prevention and control of hypertension in sub-Saharan Africa: A systematic review
Source: PLOS Glob Public Health. 2024 Jul 16;4(7):e0003459. doi: 10.1371/journal.pgph.0003459 (PMC11251591; doi:10.1371/journal.pgph.0003459)
Supplement: S1 Table — (DOCX) [file pgph.0003459.s004.docx]

**S3 Table: Effect of community-based interventions on prevention and control of hypertension**

| **Author, publication year** | **Intervention Modalities** | **Intensity & Duration** | **Main Finding description** |
| --- | --- | --- | --- |
|  |  |  |  |
| Rossouw J. E. , et al.,1993 | Exposed to a structured health education program, while further contact with the control area was avoided. Both intervention communities received a mass media program in the form of posters, billboards, mailings and items in local newspapers. In the first year of the structured program, a 4-month general awareness campaign was followed sequentially by programs aimed at (1) lowering blood pressure through regular blood pressure checks, reducing dietary salt, weight loss and medication prescribed by usual care-givers where indicated, (2) reducing blood cholesterol through lower saturated fat and cholesterol intake, (3) stress management, (4) increasing recreational physical activity and (5) smoking avoidance and cessation. During the subsequent 2 years, these themes were repeated singly and in combination, in each case using new materials and varying intensity and duration in order to maintain interest. | During the first year of structured intervention the populations were exposed to six different billboards, six posters, eight mailings, frequent news items, health messages on electricity accounts, and one special supplement in a local newspaper. During each of the remaining 2 years, the frequency of billboards, posters and mailings, was about half of the initial rate, but frequent news items and an annual special supplement in a local newspaper continued. Community-initiated activities included fun runs and family walks. Local food suppliers and restaurants were encouraged to provide low-fat, low-cholesterol and low-salt foods and display them prominently. A blood pressure station offering free blood pressure screening and educational materials was established in both intervention areas. The blood pressure stations were staffed by nursing sisters who were well-known in their communities. | Significant reductions in blood pressure, smoking, and risk score were obtained in Lll and HII alike. Total cholesterol fell by 10-12% |
|  |  | In addition to the above program, the HII communities were reached through: (1) a series of public meetings addressed by experts on each of the risk factors, (2) close liaison with community organizations, including providing speakers for their educational programs, (3) establishment of community committees to co-ordinate and encourage health activities, (4) participation in generating and testing of educational materials, (5) interpersonal small-group intervention to high-risk individuals, and (6) active follow-up of hypertensive as opposed to the passive availability of the blood pressure station in the LII area. A series of six 2-hour diet instruction sessions was held initially under the guidance of dieticians, and throughout the intervention a number of cooking competitions were held under the auspices of local organizations. A heart healthy recipe book was compiled from local entries and distributed to the entire community (also to the LII community). Stress management techniques were taught by a clinical psychologist and fitness testing and exercise training by an exercise physiologist. A number of 5-day smoking cessation seminars were held. | After intervention knowledge increased more in females from both intervention areas (and in HII males) than in the C area. The overall more favorable change in risk factors for LII and HII communities as compared to C are summarized by the change in risk scores (Table 3 and Figure 1A). Among the individual risk factors, decreases in TC were particularly large and of the same magnitude (0.26-0.31 mmol/1) in intervention and C areas. |
|  |  |  | Blood pressures and smoking decreased to a greater extent in intervention areas. |
|  |  |  | In the C area DBP in men and tobacco consumption in women actually increased over 4 years. |
|  |  |  | Community-based intervention works, and that in these particular communities a media-based health education program was more cost-effective than one which adds a greater degree of interpersonal intervention. |
| Flor et al., 2020 | Screening and diagnosis | Screening and follow- up: promoted awareness of and screening for DM and HTN in partnership with the extensive CHW network of Nightingale Hospice; conducted screenings and NCD education at community health events, targeted door -to- door campaigns, HIV and Tuberculosis (TB) support groups, retirement homes and shelters, and farms and solar plants; conducted follow- up via phone or in- person as needed, and facilitated patient visits (eg, arrange for transportation) | Barriers to healthcare and better health outcomes, including poverty, low levels of health education and limited access to affordable and nutritious food were reported across all sites. |
|  |  | Technologies for care coordination: supported the creation of a database of DM and HTN patients; enabled tablet- based data collection at community screening events and door- to- door visits | Positive views of introducing community- based NCD care and outreach services alongside requests for further expanding community services and health education initiatives ‘Outside the facility, we have adherence club where chronic patients are being taught about exercises and adherence in the community. Then we have collection points where patients fetch their medication, the collection point are at scheduled halls or education institutions. That has helped us…because we take the medication to the people.’—Frontline health worker |
|  |  | Workforce development: trained CHWs in DM and HTN screening processes; provided screening equipment to CHWs; provided 4 day trainings on DM and HTN management for clinic- based providers | Some underlying tensions in patient–provider relationships related to long wait times and lack of trust (from patients' perspective) and reluctance to make necessary changes to diet and exercise (from providers' perspective) ‘It’s not good and it’s also not bad, it’s in between. There are some patients who understanding our working conditions that maybe we have shortage of medication at that particular time and maybe we are busy because this is the only clinic in the community, but there are some patients who do not understand, they would say that we are slow or do not care about the patients.’—Facility manager |
|  |  | Clinical and non- clinical patient support: clinic nurses ensured confirmatory diagnoses and follow- up with patients who failed to attend appointments, pick up medications, or meet control targets; organized patient support groups for patient empowerment and self- care; set up gardening, via the Department of Agriculture and Forestry, and village savings and loans programs. | Role for traditional medicine alongside more formal health sector ‘If one doesn’t have cash, he can’t get to the hospital. We then use the services of prophets and traditional healers.’—Patient |
| Pastakia et al., 2013 | Home based screening and Community based screening | Community mobilizers were used to sensitize the community to the availability of a 2-day long diabetes and hypertension screening program in the upcoming week to commemorate World Diabetes Day. | The home-based screening strategy identified 13 participants (6% of the total population screened) with a SBP greater than or equal to 160mmHg, while the community-based strategy identified 35 participants (10% of the total population screened). Participants in the community based screening were almost twice as likely to have a positive screening for hypertension compared to the home based screening arm (OR=1.93, Fischer’s exact test, P=0.06). With regards to diabetes screening, 54 participants (23%) and 27 participants (8%) in the home-based screening and community-based screening, respectively, met the predefined threshold requiring confirmatory blood sugar testing. Participants in the home-based screening were 3.5 times more likely to have a positive screening result than the participants in the community-based screening (OR=3.51, Fischer’s exact test, P<0.01). |
|  | a one-day training session on hypertension and diabetes. This training highlighted the epidemiology, pathophysiology, risk factors, diagnosis/measurement and screening methods for hypertension and diabetes. Counselors were also instructed on the overall goals of treatment and prevention options. | The availability of this free screening was advertised through standard modalities of sensitization including discussion at church, via community chiefs meetings (locally referred to as Barazas), and by word of mouth via community leaders. | Both screening strategies identified a large pool of high-risk participants which had similar rates of poor follow-up after screening. When screening for hypertension is assessed in isolation from the other aspects of the healthcare system, the community-based screening strategy seems to attract higher risk participants compared to a more comprehensive home-based screening strategy. One of the major benefits of home-based screening, not specifically discussed in this study, is the potential for greater linkage to the healthcare system to facilitate home-based care in the future. |
| Van de Vijver, et al., 2016 | Awareness campaigns, household visits for screening, and referral and treatment of people with hypertension | 1. Raising awareness prior to the door-to-door campaign: Through radio jingles on the local radio station Koch FM and awareness campaigns through visits at churches, mosques, and other public spaces within the slum to create understanding of CVD and increase participation in the program. | We observed a significant reduction in mean SBP when comparing before and after measurements in both intervention and control groups, 2.75 mmHg (95% CI 4.33 to 1.18, p‑0.001) and 1.67 mmHg (95% CI 3.17 to 0.17, p‑0.029), respectively. Among people with hypertension at baseline, SBP was reduced by 14.82 mmHg (95% CI 18.04 to 11.61, pB0.001) in the intervention and 14.05 (95% CI 17.71 to 10.38, pB0.001) at the control site. However, comparing these two groups, we found no difference in changes in mean SBP or hypertension prevalence |
|  |  | 2. Improving access to screening: Through door-to door household visits by community health workers (CHWs) who measured BP and other anthropometric outcomes and provided brief counseling on cardiovascular risk factors to all consenting adults aged 35 years and above. Because earlier research revealed that unhealthy diets and reduced physical exercise, aligned with the epidemiological transition taking place in these settings, increase CVD risk; the CHWs assessed study participants’ level of engagement in risky lifestyle behavior, including tobacco use, alcohol use, physical activity levels, and dietary habits. Consequently, they provided brief counseling assistance (BCA) on healthy lifestyle modification using the six A’s approach Ask, Advice, Assist, Arrange, Agree, and Affirm (23). Traditional BCA does not include the sixth A (Affirm) as a separate entity. However, due to the importance of lifestyle change, CHWs encouraged study participants to continue with any healthy lifestyle behavior in which they were currently engaged. | The prevalence of hypertension and mean SBP were similar between the sites at baseline, but the DBP was higher in the control group than in the intervention group (81.4 mm Hg vs. 83.0 mm Hg, p 0.001). The intervention group was older, less educated, and poorer. It was comprised of more females. It had less alcohol and tobacco use but more physical activity compared with the control group. |
|  |  | 3. Facilitating access to treatment: Through distribution of vouchers for a free visit to the intervention clinic to persons identified with hypertension; health service improvements (nurses and clinical officers were trained, primary care guidelines for hypertension management developed, and equipment supplied); opening of a clinic at a central location in the slum, within walking distance for the local population, and also open on the weekends to increase access to care for daily laborers who work during weekdays; and incentives to CHWs to encourage people identified with hypertension to come to the clinic for an initial visit. | Comparing the intervention and control group, we found no significant difference in the mean SBP reduction at population level (0.32 mmHg, 95% CI 2.48 to 1.83) (Table 2). Likewise, no significant difference was detected in the DBP reduction between intervention and control (1.09 mmHg, 95% CI 0.29 to 2.46) |
|  |  | 4. Promoting long-term retention in care: Through incentives to CHWs to encourage patients to visit the clinic during the first 6 months, medication subsidies, creation of patient support groups to build knowledge and understanding through train-the-trainer sessions, and SMS reminders to improve adherence. | Among those with hypertension at baseline in the intervention (n 388) and control (n 266) settings, the reduction of SBP pre versus post was larger: 14.82 mmHg (95% CI 11.61 to 18.04) in the intervention and 14.05 (95% CI 10.38 to 17.71) at the control site. DBP decreased by 7.55 mmHg (95% CI 5.57 to 9.54 mmHg) in the intervention group and 10.67 mmHg (95% CI 8.44 to 12.89 mmHg) in the control group. In the control group, we also detected a decrease at population level in smoking (OR 0.73, 95% CI 0.56 to 0.95) and alcohol use (OR 0.71, 95% CI 0.57 to 0.88). Among patients with hypertension in the control group, smoking (OR 0.51, 95% CI 0.28 to 0.90, p 0.021) and alcohol use (OR 0.62, 95% CI 0.38 to 0.99, p 0.044) also reduced significantly. Insufficient intake of fruits and vegetables increased significantly at population level both in intervention (OR 1.30, 95% CI 1.08 to 1.56, p 0.006) and control (OR 1.42, 95% CI 1.15 to 1.76, p 0.001) settings. |
| Siedner et al., 2018 | Home based screening | In 2010, all individuals who participated in the homebased survey were also offered a physical examination to determine weight, height and blood pressure, using the WHO STEPS protocol. Blood pressure was measured using Omron automated blood pressure monitors. | Approximately one quarter (n=3074, 26.2%) of participants were found to have elevated pressure during the home-based blood pressure screening, of whom 1368 (44.5%) reported having been previously diagnosed or currently on treatment. Of those who had been previously diagnosed or in hypertension care, 1169 (85.5%) were currently on hypertension treatment. Participants who were not previously aware of their condition were significantly younger, and more likely to be men, married, employed, have a higher level of education and be living in periurban areas than those who had been previously diagnosed or on treatment |
|  |  |  | we found strong evidence that women (OR=2.76, 95% CI 1.97 to 3.88, p<0.001) and those of older age (OR=12.89, 95% CI 6.62 to 25.11, p<0.0001, comparing those 45–59 years vs those <30) were more likely to present to hypertension care within 2 years of home-based diagnosis |
| Kotwani et al., 20141 | - education, - screening - referral appointment to a health facility and - Transport voucher. | The linkage to care intervention included education, referral appointment to a health facility and transport voucher. Each participant received an individualised counselling session with an experienced nurse, which included education about the chronic nature of hypertension, possible complications of untreated disease, need for lifestyle modifications and potential necessity for lifelong medications. Each session lasted 5–10 min and was performed by the same nurse for all subjects. Participants were then given a referral appointment to the nearest local health centre (Bwizibwera Health Center IV) or the regional hospital (Mbarara Regional Referral Hospital) if hypertensive urgency was diagnosed (systolic BP ≥180 mmHg or diastolic BP ≥110 mmHg). Appointments were scheduled 3–30 days after screening. | Within 6 months, 178 (83%) of the 214 participants who received the linkage intervention (the study cohort) visited a health facility for hypertension management. Independent predictors of successful linkage included older age, female gender, higher education, manual employment, tobacco use, alcohol consumption, hypertension family history and referral to local vs. regional health centre. Barriers for patients who did not see care included expensive transport (59%) and feeling well (59%). Participants ≥60 years were more likely to link than those aged 18–29 years (RD = 36.9%, 95% CI 15.7, 58.2). There was no statistically significant difference in linkage between those aged 18–29 years, 30–44 years and 45–59 years. Women had a 14.4% greater probability of linking than men (RD = 14.4%, 95% CI 5.2, 23.7). Increasing level of education was associated with an increase in the likelihood of successful linkage. Compared to those with no formal education, persons with tertiary education or beyond were the most likely to link |
|  |  |  | Feeling well (59%), expensive transportation (59%), transportation difficulty/inconvenience (33%), fear of being reprimanded by the clinic staff for missing a scheduled appointment (26%), family obligations (22%) and responsibilities at work (22%) were the most common barriers for not linking to care (Table 3). Notably, no participant cited stigma as a barrier in open-ended interview questions. |
| Nikkil Sudharsanan et al., 2020 | Home-based screening & diagnosis | Informing people in the household that their blood pressure was high; that high blood pressure can have adverse health consequences if left uncontrolled and that they should seek further care. | Home –based hypertension screening resulted 4.7 mmhg reduction in SBP for soth Africa women. |
